# Transcriptome Profiling to Dissect the Role of Genome Duplication on Graft Compatibility Mechanisms in Watermelon

**DOI:** 10.3390/biology11040575

**Published:** 2022-04-11

**Authors:** Mohamed Omar Kaseb, Muhammad Jawad Umer, Muhammad Anees, Hongju Zhu, Shengjie Zhao, Xuqiang Lu, Nan He, Eman El-Remaly, Ahmed El-Eslamboly, Ahmed F. Yousef, Ehab A. A. Salama, Abdulwahed Fahad Alrefaei, Hazem M. Kalaji, Wenge Liu

**Affiliations:** 1 Zhengzhou Fruit Research Institute, Chinese Academy of Agricultural Sciences, Henan Joint International Research Laboratory of Fruits and Cucurbits Biological Science in South Asia, Zhengzhou 450009, China; mohamedkaseb@yahoo.com (M.O.K.); umermjawad@yahoo.com (M.J.U.); aneesgscaas@outlook.com (M.A.); zhuhongju@caas.cn (H.Z.); zhaoshengjie@caas.cn (S.Z.); luxuqiang123@163.com (X.L.); henan@caas.cn (N.H.); 2 Cross Pollenated Plants Department, Horticulture Research Institute, Agriculture Research Center, Giza 12119, Egypt; emanelrmaly@yahoo.com (E.E.-R.); azaz2005asd@yahoo.com (A.E.-E.); 3 State Key Laboratory of Cotton Research, Chinese Academy of Agricultural Sciences, Anyang 455000, China; 4 Department of Horticulture, College of Agriculture, Al-Azhar University (Branch Assiut), Assiut 71524, Egypt; ahmed.yousuf@azhar.edu.eg; 5 Agricultural Botany Department, Faculty of Agriculture (Saba Basha), Alexandria University, Alexandria 21531, Egypt; ehabsalama89@alexu.edu.eg; 6 Department of Zoology, College of Science, King Saud University, P.O. Box 2455, Riyadh 1145, Saudi Arabia; afrefaei@ksu.edu.sa; 7 Department of Plant Physiology, Institute of Biology, Warsaw University of Life Sciences SGGW, 02-787 Warsaw, Poland; hazem@kalaji.pl; 8 Institute of Technology and Life Sciences–National Research Institute (ITP), 05-090 Raszyn, Poland

**Keywords:** WGCNA, diploid, tetraploid, IAA and ZR signal, pathways, hormones antioxidants (AOX)

## Abstract

**Simple Summary:**

Triploid seedless watermelon cultivars have high demand globally, and they are excellent in quality compared to diploid seeded watermelons. A low number of seedlings are produced as a result of grafting in triploid and tetraploid watermelons. In this regard, to understand the influencing factors of genome duplication on graft compatibility, we performed a comparative transcriptome analysis between tetraploid and diploid watermelons grafted on squash rootstock with the splice method. A weighted gene co-expression network analysis (WGCNA) was performed using the common differentially expressed genes (DEGs) between diploid and tetraploid plants of watermelon grafted seedlings and the contents of hormones antioxidants (AOX), sugars, and starch at 0, 3, and 15 days after grafting (DAG). Higher survival rates and contents of hormones, AOX, sugars, and starch were observed in tetraploid grafted seedlings compared to diploid ones. We concluded that genome duplication significantly affected gene expression in the IAA and ZR signal transduction and AOX biosynthesis pathways in the grafted plants, resulting in the regulation of hormone levels’ signal pathways, promoting plant survival. These genes are identified for the first time, and no previous reports about their role or functions in watermelon are available.

**Abstract:**

Watermelon (*Citrullus lanatus*) is a popular crop worldwide. Compared to diploid seeded watermelon, triploid seedless watermelon cultivars are in great demand. Grafting in triploid and tetraploid watermelon produces few seedlings. To learn more about how genome duplication affects graft compatibility, we compared the transcriptomes of tetraploid and diploid watermelons grafted on squash rootstock using a splicing technique. WGCNA was used to compare the expression of differentially expressed genes (DEGs) between diploid and tetraploid watermelon grafted seedlings at 0, 3, and 15 days after grafting (DAG). Only four gene networks/modules correlated significantly with phenotypic characteristics. We found 11 genes implicated in hormone, AOX, and starch metabolism in these modules based on intramodular significance and RT-qPCR. Among these genes, two were linked with IAA (r^2^ = 0.81), one with ZR (r^2^ = 0.85) and one with POD (r^2^ = 0.74). In the MElightsteelblue1 module, *Cla97C11G224830* gene was linked with CAT (r^2^ = 0.81). Two genes from the MEivory module, *Cla97C07G139710* and *Cla97C04G077300*, were highly linked with SOD (r^2^ = 0.72). *Cla97C01G023850* and *Cla97C01G006680* from the MEdarkolivegreen module were associated with sugars and starch (r^2^ = 0.87). Tetraploid grafted seedlings had higher survival rates and hormone, AOX, sugar, and starch levels than diploids. We believe that compatibility is a complicated issue that requires further molecular research. We found that genome duplication dramatically altered gene expression in the grafted plants’ IAA and ZR signal transduction pathways and AOX biosynthesis pathways, regulating hormone levels and improving plant survival.

## 1. Introduction

Watermelon (*Citrullus lanatus* L.) is an essential and popular summer fresh fruit worldwide [1]. Triploid seedless watermelon cultivars are the most desired by consumers, with a high price and more excellent quality than seeded watermelons [2]. Seedless watermelons are triploids (3n = 33), produced by crossing a tetraploid (4n = 44) as a female parent with a diploid (2n = 22) as a male parent [3,4]. Tetraploid plant induction can be achieved by different methods, such as applying aqueous chemicals solution, viz., Colchicine and Oryzalin to the apical meristem of diploid seedlings [5,6,7].

Polyploidy, the presence of more than two genomes in a nucleus or cell [8,9], is a common phenomenon among plants and is usually related to fitness and is well-adapted [10]. One of the core advantages of polyploidy in the watermelon is triploid (seedless) fruits; another benefit of gene redundancy is diversifying gene function by altering redundant copies of essential genes [10,11,12]. Interestingly, after genome doubling, the artificially induced autopolyploids always exhibit new characters, such as increased DNA contents, high secondary metabolite substances, large tissues and organs, improved yield, or higher contents of chlorophyll, lycopene, fructose, and glucose [3,4,6,13,14]. Additionally, other effects have been observed such as increased quality hardiness [15,16,17] and higher tolerance to both abiotic and biotic stresses when compared to diploids in watermelon [18], rice [19,20], citrus [21], black locust [22], honeysuckle [23], kinnow mandarin [24], cotton [25], and rangpur lime [26]. In this respect, grafting is another technique frequently used in horticultural crops. Commercially, vegetable grafting is employed due to its potential to provide a wide range of essential benefits, such as enhancing resistance to biotic and abiotic stresses and improving and promoting plant growth [27], in addition to other advantages. In particular, watermelon is known to achieve high compatibility when grafted with cucurbit hybrids [28].

Recently, with the wide adoption and implementation of various omics technologies, it has become easier to understand the compatibility mechanisms and identification of candidate genes. The transcriptome is an organism’s whole collection of transcripts, and transcriptomics is the study of gene expression at the RNA level [29]. Li et al. [30] discovered that in apple shoots grafted onto a dwarf rootstock, the expression of the polar auxin transport-related gene PIN1 was significantly downregulated, implying that the change in gene expression reduced polar transport of indole-3-acetic acid (IAA) from the top-down, resulting in an insufficient supply of IAA to the apple roots. The expression levels of ARF1, ARF8, GH3, and IAA4 were discovered to be adversely linked with growth vigor and IAA content by Liu et al. [31]. The differential expression of KO1 and GA2OX1 in grafted plants also impacted GA metabolism. Furthermore, most antioxidant enzyme genes were upregulated in red tangerine tree leaves, resulting in increased peroxidase activity. Furthermore, investigations of gene expression variations in different scion–rootstock combinations of sweet cherry [32] and grape [33] have been conducted.

Graft compatibility is a complex process involving anatomical and biochemical interactions [34,35] that starts with wound response, callus formation, and eventually, the creation of a cambium functional vascular system between rootstock and scion [36]. These processes may decide a grafted plant’s destiny [37]. However, no clear knowledge of how these therapies impact incompatibility reactions exists [38]. According to Melnyk [39], who explained the graft junction formation, steps during the first 1–2 days after grafting are as follows: (1) adhesion of scion and stock (2) cell divisions are initiated and there is callus formation (3) callus makes contact between scion and stock. After one week or more, (4) calluses differentiate into xylem and phloem elements, (5) vascular connections are re-established, and cytokinins play an essential role in regulating scion/stock interactions [40] and cytokinin is required for wound healing and vascular regeneration in the graft region area [41,42]. Graft incompatibility in fruit trees occurs due to hormone imbalance [43,44,45,46]. One of these plant hormones, auxin, causes cell elongation in shoots and regulates plant growth. Low auxin concentrations promote intact roots, whereas higher concentrations inhibit growth [47,48], such as in maize roots [49]. Tobacco cytokinin promotes root development by increasing the size of the root apical meristem.

Similarly, mutations that partly alter cytokinin perception promote root development and speed up the vascular differentiation process in the root tip [48]. The buildup of reactive oxygen species (ROS) causes oxidative damage and cell death [18,50,51]. As a result, boosting the activity of defense-related enzymes such as catalase (CAT) and peroxidase (POD) may help plants scavenge ROS and improve their resilience to a variety of stressors [1,52]. POX and CAT convert hydrogen peroxide (H_2_O_2_) to H_2_O [53]. The graft union formation coincides with AOX activities in dealing with oxidative stress [54,55]. In comparison, incompatible grafts showed higher H_2_O_2_ levels and lower AOX activities in the grafting region [54,56,57]. On the other side, POD and CAT activities were increased in the grafted plants [18,55]. Furthermore, carbohydrates play an essential role in plants’ cellular activities by providing energy [58,59]. Graft union development is positively correlated with the content of carbohydrates present in scion and rootstock at grafting time, higher carbohydrates levels, and auxins and cytokinin, essential for effective callus formation. Importantly, interactions among rootstock and scion cultivars for utilizing carbohydrates can influence starch depletion throughout callus development; the survival rate increases by increasing starch content [59,60].

Previous studies have focused on studying compatibility by comparing different rootstocks with incompatibility differences [45,61]. To the best of our knowledge, no research has been conducted before on polyploidy in plant grafting. Concerning this study, we compared diploid and tetraploid watermelon plants, which have significant differences in survival rate, to study the effect of genome duplication in graft compatibility at physiological and molecular levels, which leads to a good explanation of the compatibility mechanisms. Furthermore, this is first comparison of polyploid watermelons in terms of graft compatibility.

## 2. Materials and Methods

### 2.1. Plant Material

Autotetraploid watermelons were induced artificially by colchicine from a homozygous diploid watermelon, then crossbred with a diploid watermelon to create an autotriploid watermelon as well as a squash interspecific hybrid (Xijiaqiangsheng), which is widely used in China as a rootstock, which was obtained by crossing a diploid watermelon with autotetraploid watermelon—Zhengzhou Fruit research institute, CAAS China.

### 2.2. Grafting Methods, Growth Conditions, and Sampling

The splice method was chosen based on the results of our previous research [62]. After using the splice grafting method, significant differences between diploid and tetraploid plants in the previous study [62] were recorded. Plants were cultivated and grafted in a glass house, keeping the temperature between 25 and 30 °C with 60–85% relative humidity. THtool-V151 En (Campbell Scientific Ltd., Beijing, China) was used to record temperature and humidity at each experimental plot’s center.

Samples (grafting union) were taken as three biological replicates to determine hormones, AOX, sugars and starch. Each biological replicate sample from 30 plants was collected at three different stages, 0, 3, and 15 days after grafting (DAG). Total samples were collected at 0, 3, and 15 days after grafting. We collected 30 plants and considered them as one replicate. Samples at 0 days after grafting were considered as controls [63].

### 2.3. Determination of Survival Rate

Survival rates were recorded after 15 days of grafting by counting the successful seedlings and dividing them by the total number of the grafting seedlings using the following formula [1,64].

$$\mathrm{Survival}\;\mathrm{rate}=\frac{\mathrm{Number}\;\mathrm{of}\;\mathrm{survived}\;\mathrm{grafted}\;\mathrm{plants}}{\mathrm{Total}\;\mathrm{number}\;\mathrm{of}\;\mathrm{grafted}\;\mathrm{plants}}\;\times \;100$$

### 2.4. Phytohormone Determinations

Diploid and tetraploid plant graft unions from three biological replicates were used to extract Indole-3-acetic acid (IAA) and Zeatin Riboside (ZR). We used the ELISA method proposed by Mo et al. [40] to determine the presence of the antigen.

### 2.5. Assay for Antioxidant Enzymes’ Activity and H_2_O_2_ Contents

The capacity of superoxide dismutase (SOD) activity in the graft union sample supernatant to prevent photochemical reduction of nitro blue tetrazolium (NBT) was measured by measuring the absorbance at a 560 nm wavelength using the SOD assay kit/YX-C-A500. The CAT test kit/BC0200 was also used to assess catalase activity by measuring the rate of change in H_2_O_2_ absorbance in 60 s at 240 nm using a spectrophotometer. POD can catalyze the oxidation of phenols and amines by H_2_O_2_, which has the dual function of eliminating the toxicity of H_2_O_2_, phenols, and amines. POD catalyzes the oxidation of specific substrates by H_2_O_2_ and has characteristic light absorption at 470nm when using the POD assay kit/YX-C-A502. Using the H_2_O_2_ test kit/YX-C-A400 (Sino best biological technology co, Beijing, China) according to the manufacturer’s instructions, H_2_O_2_ produces a yellow titanium peroxide complex with titanium sulphate, with distinctive absorbance at 415 nm wavelength. For each duplicate, three biological replications from grafting unions at 0, 3, and 15 DAG were obtained from three distinct plants.

### 2.6. Quantification of Sugars and Starch

To estimate starch content, the starch assay kit/YX-C-C400 was used, and for sugar content estimation, the sugars assay kit/YX-C-B629 was used (Beijing, China: Sino Best Biological Technology Co., Ltd.) according to the manufacturer’s instructions. For each replicate, three biological replications from grafting unions at 0, 3, and 15 DAG were obtained from three distinct plants.

### 2.7. Construction and Sequencing of the RNA-Sequencing Library

A previously reported approach [65] was used to create the RNA-Seq library. To begin, total RNA was extracted from samples using the TIANGEN kit (Beijing, China), and contamination and degradation of RNA were checked using a 1% agarose gel. Then, using a Nano Photometer^®^ spectrophotometer, the purity of RNA was determined (IMPLEN, CA, USA). After that, a Qubit^®^ RNA Assay Kit and a Qubit^®^ 2.0 Fluorometer were used to estimate RNA concentration (Life Technologies, CA, USA). Finally, an Agilent Nano 6000 test kit was used to assess RNA integrity (Santa Clara, CA, USA).

Sequencing libraries were created using the NEBNextR UltraTM RNA Library Prep Kit for Illumina R (NEB, USA) and sequenced on an Illumina HiSeq platform, and 125-bp/150 bp paired-end reads were obtained. After the libraries’ detection, sequencing was performed on the Illumina HiSeq platform v3.6. Raw readings were filtered to acquire excellent-quality reads. Data were extensively examined to detect any erroneous readings; moreover, GC content was also monitored, so that clean reads were acquired and could be utilized for the remaining stages. Hisat2, v2. 1. 0 was used to sequence clean reads using the reference genome (97103 V2) 4. Transcripts or gene expression levels were assessed by calculating FPKM (fragments per kilobase of transcript per million fragments mapped) [66]. Deseq2 v1.30.1 was used to investigate differential gene expression across different samples. After that, multiple hypothesis tests were utilized to correct the hypothesis test probability (*p*-value) by the Benjamini–Hochberg method to produce the false discovery rate (FDR), and the Bioconductor package Custer-Profller v3.14 was used for KEGG enrichment analysis.

### 2.8. Mapping to Watermelon Genome V2 and Quantification of Gene Expression

TopHat v2.0.12 was used to match clean reads to the watermelon reference genome (http://cucurbitgenomics.org/organism/V1, accessed on 14 March 2022). HTSeq v0.6.125 was used to count reads in features (genes in this example). The FPKM values were calculated using gene lengths and read counts assigned to genes [66].

The DESeq R package [67] was used to identify DEGs between diploid and tetraploid plants, and Benjamini–Hochberg-adjusted P-values of 0.05 were deemed statistically significant [68,69].

### 2.9. Enrichment Analysis of DEGs and Weighted Gene Coexpression Network Analysis (WGCNA) for Identifying Correlated Gene Networks

DEG enrichment in Kyoto Encyclopedia of Genes and Genomes (KEGG) pathways was tested using the KOBAS program v 2.0 [70]. To simplify genes into co-expressed modules, WGCNA was performed in R with default settings [71,72]. An adjacency matrix was created using normalized FPKM data. Using the default parameters, correlation-based connections between phenotypes and gene modules were estimated using the phenotypic data input into the WGCNA package v1.70-3. The adjacency matrix was converted into a topological overlap matrix using the WGCNA package (TOM). Transcripts with similar expression patterns were combined into one module once the network was built, and eigengenes were determined for these modules as well. The genes from each module were exported using Cytoscape’s default settings.

### 2.10. Validation of Intramodular Candidates through RT-qPCR Analysis

For each sample, three independent biological replicates and three technical replicates were used to analyze gene expression using RT-qPCR [73]. The total RNA was extracted using the TIANGEN kit (Beijing, China). The Takara kit (Tokyo, Japan) containing reverse transcriptase was used to make cDNA. The target candidate genes for the gene modules were chosen based on intramodular gene significance and annotation data from the watermelon reference genome database (http://cucurbitgenomics.org/organism/V1, accessed on 14 March 2022). The primer design and RT-qPCR conditions were performed as previously described [74]. Actin (*Cla016178*) was used as a reference control gene [75]. The internet program “Primer 3 v4.1.0” (https://primer3.ut.ee/, accessed on 14 March 2022) was used to build primers for RT-qPCR (Supplementary Data). The amplicon (PCR product) ranges were set between 80 and 200 bp. To increase the product, a Roche Light-Cycler 480 II was employed [74,76].

### 2.11. Statistical Analysis

Using SPSS 18.0 Statistics, the data were submitted to analysis of variances (SPSS Inc., Chicago, IL, USA). Duncan’s multiple range test was used to assess the differences between treatment means at a 0.05 probability level. OriginPro 8.5 was used to create the graphics (OriginLab Corp., Northampton, MA, USA).

## 3. Results

### 3.1. Survival Rates of Diploid and Tetraploid Watermelon Grafted by Splice Method

**Figure 1:**
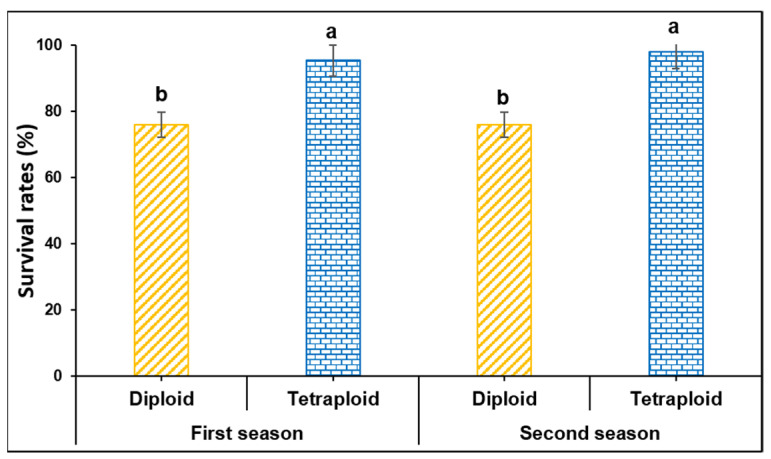
Plant survival rates in diploid and tetraploid plants of watermelon at 15 DAG during March and August 2019 cropping seasons. The bars with different letters differ significantly (*p* < 0.05), and columns indicate mean ± SE.

Plant survival rates were recorded in diploid and tetraploid plants after grafting during two consecutive seasons, March and August 2019. They were 75.97 and 95.23% in the first season and 75.87 and 97.83% in the second season, respectively (Figure 1).

### 3.2. Measurement of IAA and ZR in the Grafting Union among Diploid and Tetraploid Plants of Watermelon during the Grafting Process

**Figure 2:**
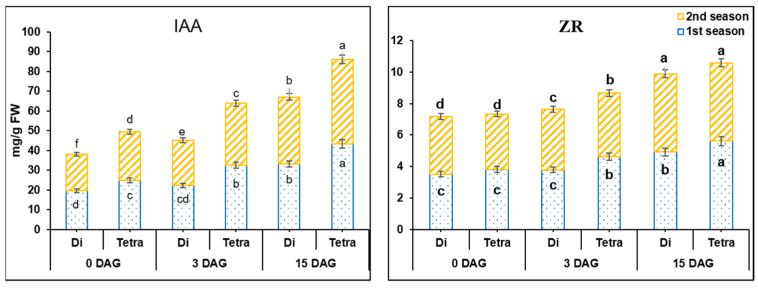
IAA and ZR hormones’ contents in diploid (Di) and tetraploid (Tetra) plants of watermelon at 0, 3, and 15 DAG during March and August 2019. The bars with different letters show significant differences (*p* < 0.05), and columns indicate mean ± SE. A comparison is made between diploid and tetraploid plants over 0, 3, and 15 DAGs.

Hormone contents and increment rates in tetraploids were significantly different at the *p* < 0.0.5 level from diploids during the grafting process, especially 2–3 days after grafting (DAG) (Figure 2).

At 0 DAG, IAA contents in tetraploid were 1.28- and 1.32-fold higher than diploids in the first and second season, respectively (Figure 2). Additionally, the increment rate of IAA in the graft union at 3 DAG showed a higher increase in tetraploid than in diploid plants; it was 14.48 and 30.29% in the first and 21.97 and 27.32% in the second season in diploid and tetraploid plants, respectively. The increment rates at 15 DAG were 48.6 and 33.33% in the first and 48.55 and 36.62% in the second season in diploid and tetraploid plants of watermelon plants, respectively.

ZR results showed insignificant differences between diploid and tetraploid plants at 0 DAG, while at 3 and 15 DAG, there were highly significant differences during both seasons (Figure 2). ZR contents at 3 DAG were increased by 8.17 and 21.52% in the first season, and 5.32 and 14.77% in the second season in diploid and tetraploid plants, respectively. The increment rate at 15 DAG was 29.26 and 21.12% in the first season, and 28.99 and 23.35% in the second season in diploid and tetraploid plants of watermelon, respectively.

### 3.3. Measurement of POD, SOD, CAT, and H_2_O_2_ Contents in the Grafting Union among Diploid and Tetraploid Plants of Watermelon

**Figure 3:**
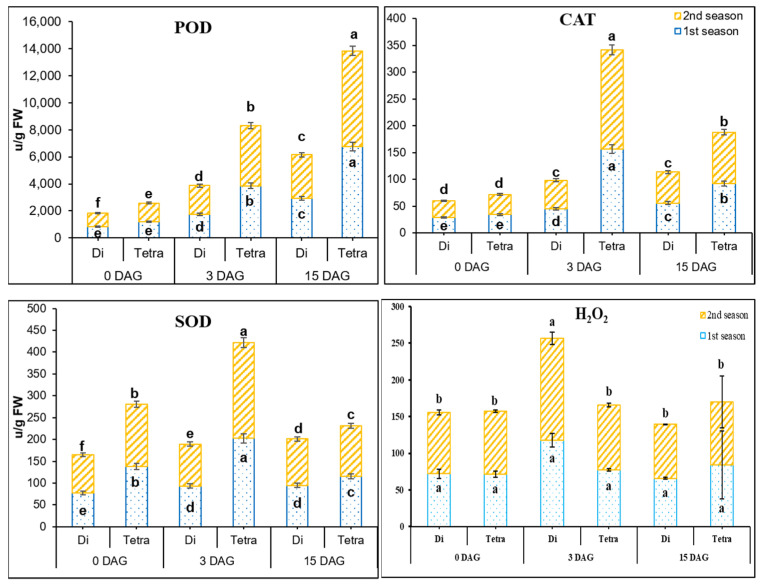
POD, SOD, CAT, and H_2_O_2_ contents in diploid (Di) and tetraploid (Tetra) watermelons grafted with splice method at 0, 3, and 15 DAG during March and August 2019 cropping seasons. The bars with different letters differ significantly (*p* < 0.05), and columns indicate mean ± SE.

Antioxidant activities were significantly different between diploid and tetraploid plants of watermelon during the grafting process in both seasons (Figure 3). The highest POD activities were shown in tetraploid at 0 DAG, i.e., 1.41- and 1.42-fold, compared to diploids, in the first and second season, respectively. On the other hand, POD activities’ increment rates at 3 DAG, were 108.07 and 223.53% in the first, and 116.71 and 219.13% during the second season, in diploid and tetraploid plants of watermelon, respectively.

Tetraploids had significantly greater CAT levels than diploids (Figure 3); at 0 DAG, tetraploid CAT activities were 1.2- and 1.2-fold higher than diploids in the first and second seasons, respectively. CAT activities in the graft union, on the other hand, increased by 57 and 354.8% in the first season, and 70.1 and 400% in the second season in diploid and tetraploid watermelon plants, respectively, at 3 DAG.

Higher SOD activity was observed in tetraploid than diploid watermelons (Figure 3); at 0 DAG, SOD activities in tetraploids were 1.79- and 1.63-fold higher than diploids in the first and second season, respectively. At 3 DAG, SOD activities were increased in tetraploids compared to diploids by 20.57 and 46% in the first season, and by 10.2 and 53.66% in the second season.

H_2_O_2_ accumulation leads to cell death. There were no significant differences between diploid and tetraploid plants in terms of H_2_O_2_ contents at 0 DAG in both seasons (Figure 3). However, at 3 DAG, the H_2_O_2_ contents in the graft union started to increase, by 64.1 and 8.59% in the first season and 65.57 and 2.33% in the second season in diploid and tetraploid plants, respectively. It was observed that the increase in H_2_O_2_ in tetraploids was much lower than in diploids; this may be because of the higher activities of antioxidants in tetraploids than diploid.

### 3.4. Sugars’ and Starch Contents in the Grafting Union among Diploid and Tetraploid Plants of Watermelon during the Grafting Process

**Figure 4:**
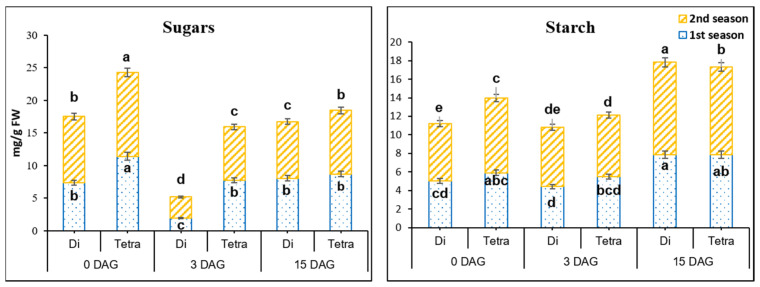
Sugar and starch contents in diploid (Di) and tetraploid (Tetra) watermelon at 0, 3, and 15 DAG. The bars with different letters differ significantly (*p* < 0.05), and columns indicate mean ± SE.

Carbohydrates supply energy to the plant, and cotyledons are the source of carbohydrates in seedlings; grafting survival rates were positively connected with an increase in starch content. At 0 DAG, there were substantial changes in sugar concentration between diploid and tetraploid watermelon plants (Figure 4). Sugar concentration was 1.54- and 1.27-fold higher in tetraploids then in diploids in in the first and second seasons, respectively. At 3 DAG, the contents of sugars decreased by 288.5 and 47.7% in the first season and 288.5 and 57.15% in the second season in diploid and tetraploid plants, respectively. It was observed that the decrement rate was significantly lower in tetraploid than diploid plants at 3 DAG. On the other hand, sugar contents at 15 DAG started to increase by 322.29 and 12.51% in the first season and 164.91 and 18.83% in the second season in diploid and tetraploid plants, respectively.

In the first season, starch contents (Figure 4) at 0 DAG showed significant differences between diploid and tetraploid plants of watermelons. Starch contents started to decrease at 3 DAG in diploid and tetraploid plants. The decrement rates in tetraploids were less than diploids by 13.87 and 7.38% in the first season, and 3.47 and 20.9% in the second season. On the other hand, at 15 DAG, starch contents increased significantly by 77.85 and 43.42% in the first season, and 56.04 and 41.68% in the second season in diploid and tetraploid plants, respectively.

### 3.5. Principal Component Analysis (PCA)

**Figure 5:**
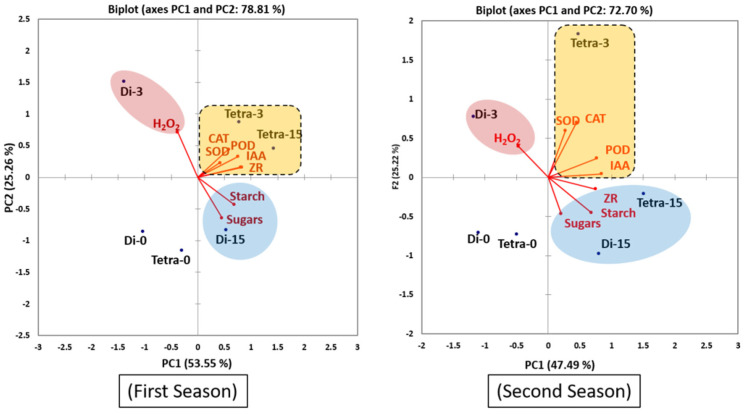
PCA scatterplot showing total AOX, hormones, starch, and sugar concentrations in diploid and tetraploid watermelon plants at three stages as a function of grafting combination (0, 3, 15 DAG). Tetra refers to a tetraploid plant, whereas Di refers to a diploid plant.

The PCA scatterplot (Figure 5) showed a clear cluster for AOX, hormones, sugars, starch and H_2_O_2_ with diploid watermelon at 3 DAG. Other clustered groups showed additional separation in more than one group, in the second, third and fourth quarters of the centroid, respectively, for diploid and tetraploid plants at different time points (0, 3, 15 DAG).

These results indicate that increase in hormones and antioxidant enzymes at 3, 15 DAG was similar in tetraploid plants and just at 15 DAG in diploid plants. While in Di at 3 DAG there was a high correlation with H_2_O_2_ which leads to cell oxidation and damage.

### 3.6. Genome-Wide Transcriptomic Analyses of Diploid and Tetraploid Plants of Watermelon

We obtained 178.49 Gb of clean data after RNA sequencing of 27 samples collected from diploid and tetraploid plants of watermelon at 0, 3, and 15 DAG, with at least 5.81 Gb clean data for each sample. Each sample has more than 93.65% of bases with a score of Q30 and above (Table 1).

The data were cleaned and mapped to the reference genome (http://cucurbitgenomics.org/organism/V1, accessed on 14 March 2022), with a mapping ratio that ranges from 6.89 to 95.96%. The outcomes of the mapping, alternative splicing prediction, genetic structure optimization, and new gene identifications were all investigated. Gene expression analysis was carried out based on the alignment findings. Functional annotation and enrichment analysis were conducted on differentially expressed genes based on their expression levels in various samples.

Between diploid and tetraploid watermelon plants, a total of 14,586 DEGs were discovered. We compared and analyzed DEGs between diploid and tetraploid watermelon plants at three key grafting stages: 0 days after grafting (DAG) (Diploid-0DAG vs. Tetraploid-0DAG), 3 DAG (Diploid-3DAG vs. Tetraploid-3DAG), and 15 DAG (Diploid-15DAG vs. Tetraploid-15DAG), using 3178 and 2002 DEGs. At three important grafting phases, 3945 genes were considerably upregulated, and 3692 genes were significantly downregulated in diploids compared to tetraploids (Table 2).

**Figure 6:**
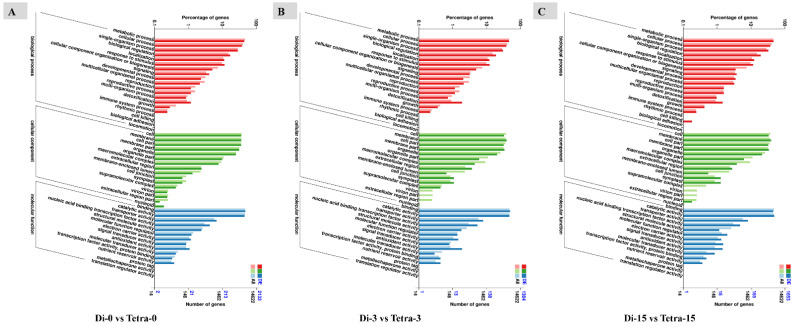
GO classification results of differentially expressed genes found by pairwise comparisons among different diploid and tetraploid plants’ grafting stages. (**A**) Diploid vs. tetraploid at 0 DAG, (**B**) diploid vs. tetraploid at 3 DAG, (**C**) diploid vs. tetraploid at 15 DAG.

To investigate gene activities linked with graft compatibility in polyploid watermelon, researchers used Gene Ontology (GO) enrichment analysis of DEGs. The most enriched GO terms in the biological process “cellular component,” and “molecular function” groups were the same for both up- and down-regulated genes: “metabolic process” and “cellular process” in the biological process category; “cell membrane,” “cell part,” and “membrane part” in the cellular components category; “binding activity” and “catalytic activity” in the molecular function category; and “binding activity” and “catalytic activity” in the mole category (Figure 6).

**Figure 7:**
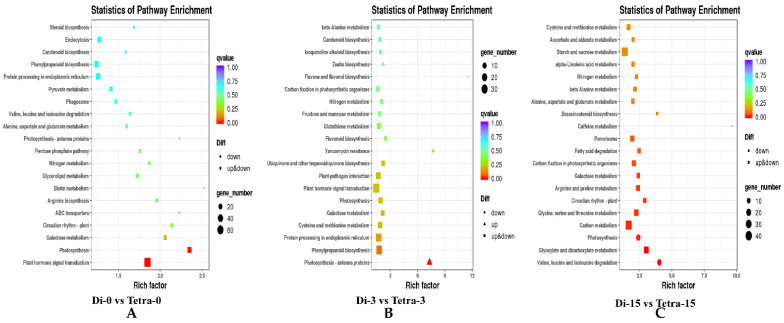
KEGG enrichment analysis of DEGs found by making pairwise comparisons among different diploid and tetraploid plants grafting stages. (**A**) Diploid vs. tetraploid at 0 DAG, (**B**) diploid vs. tetraploid at 3 DAG, (**C**) diploid vs. tetraploid at 15 DAG.

We also used a KEGG enrichment analysis to figure out the function of the DEGs. As a consequence, 1432 upregulated genes and 1147 downregulated genes were found to be concentrated in 55 metabolic pathways, which were divided into three categories: biological processes, cellular components, and molecular function. Plant hormone signal transduction is the secondary metabolic route with the most DEGs among these groups. Carbon metabolism, starch metabolism, and sucrose metabolism are all part of the metabolic categorization category in the environmental information process category. The metabolism category contains the most KEGG pathways in this study, implying that differentially expressed genes involved in metabolic pathways may play a role in watermelon graft compatibility variation (Figure 7).

### 3.7. WGCNA for the Identification of Highly Connected “Hub Genes” and Correlated Gene Modules

WGCNA is an omics analysis tool that defines itself as a network that connects all variables continuously and clusters the most strongly co-expressed variables into inflexibly specified modules. We created a transcriptome dataset of diploid and tetraploid plants at three important grafting process stages: 0, 3, and 15 DAG, and input FPKM values into WGCNA. As a consequence of the connection and co-expression patterns of different genes, 73 unique gene modules were discovered. These gene modules are color-coded and pre-presented as a network heatmap. In addition, for the WGCNA analysis, the contents of IAA, ZR, AOX, starch, and sugars at each grafting stage were employed as phenotypic data.

### 3.8. Module Analysis Based on WGCNA

**Figure 8:**
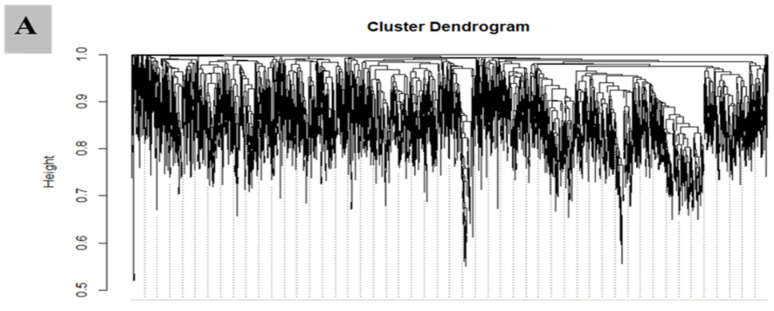

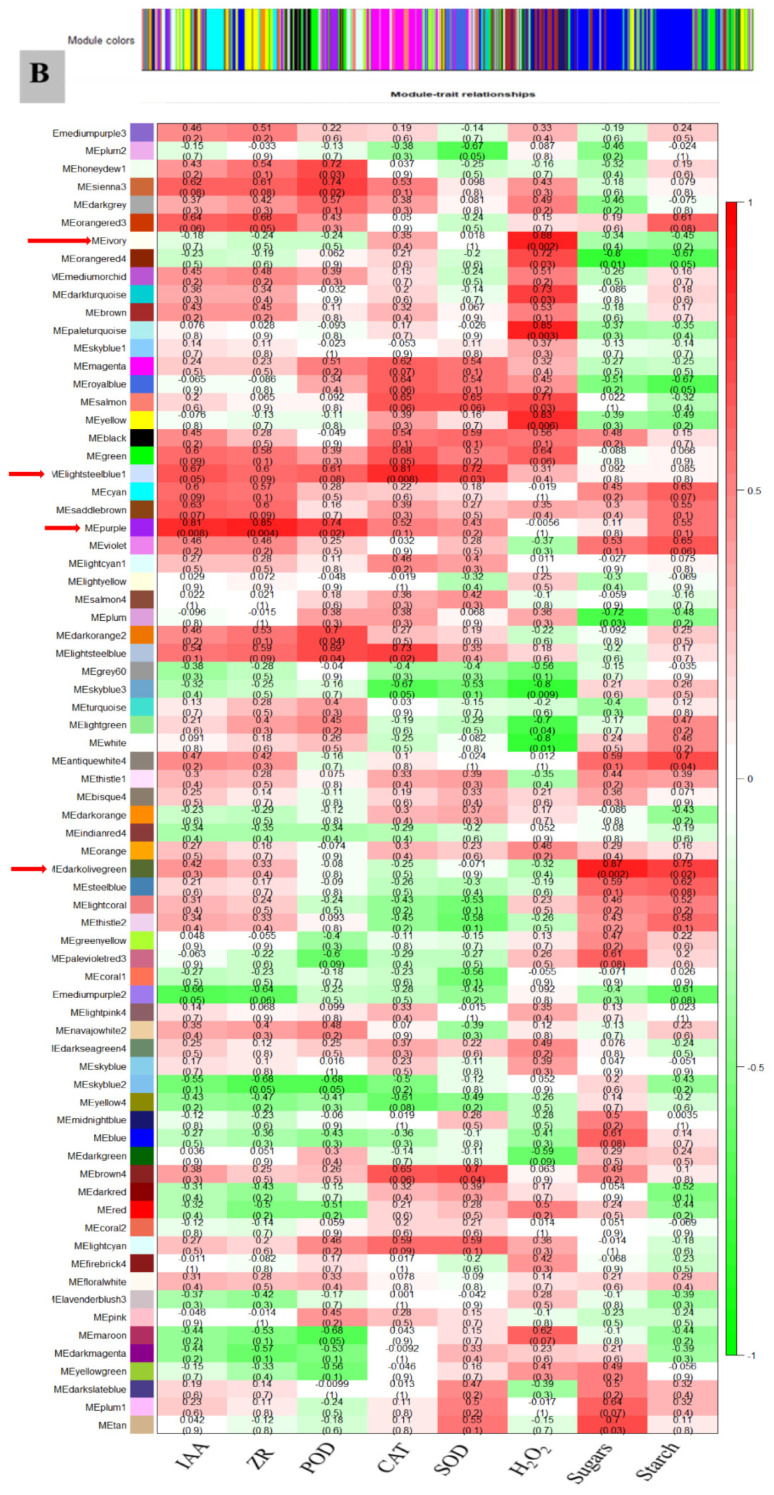
Gene cluster dendrogram in diploid and tetraploid plants based on co-expression network analysis. (**A**) Pearson correlations-based module–trait relationships. From green to red, the colors reflect r^2^ values ranging from −1 to 1. (**B**) Hierarchical clustering displaying 73 modules with co-expressed genes. Each leaflet in the tree represents a distinct gene.

Out of 73 gene modules, only 4 revealed a significant correlation with phenotypic traits. The MEpurple module comprises 331 genes and showed significant associations with IAA, ZR and POD with correlation coefficient (r^2^) values of 0.81, 0.85, and 0.74, respectively. The MElightsteelblue1 module with 21 genes was strongly correlated with CAT and SOD, with r^2^ = 0.81 and 0.72, respectively. MEivory module consisting of 20 genes recorded a significantly high correlation with H_2_O_2_ (r^2^ = 0.88). The MEdarkolivegreen module consisting of 35 genes showed a significantly high correlation with sugars and starch (r^2^ = 0.87, and 0.75). A detailed description of gene module and trait correlations is presented in (Figure 8A,B).

**Figure 9:**
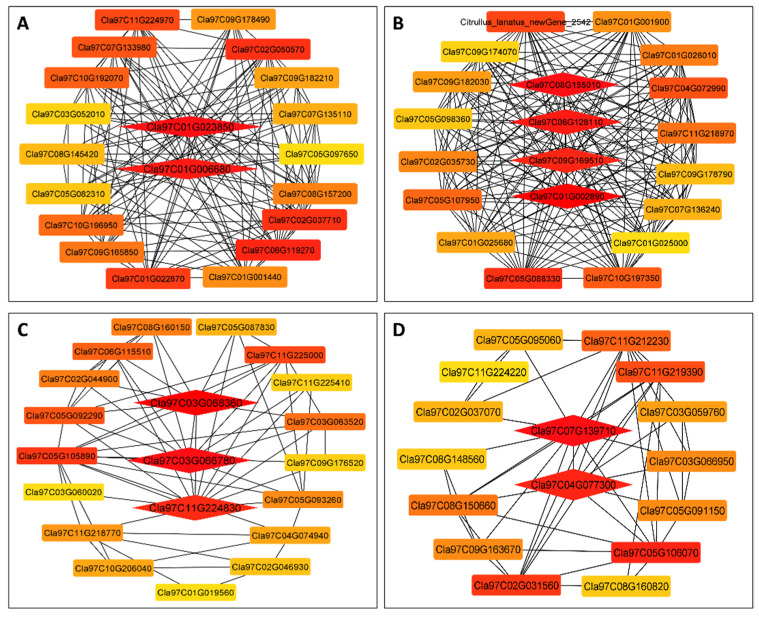
Network visualization of hub genes (**A**) MEdarkolivegreen (**B**) MEpurple (**C**) MElightsteelblue1 (**D**). ME-ivory module gene. The red highlighted cells indicate the hub genes.

These putative candidate genes are highlighted in red in the gene networks; these gene networks’ clear features can easily be observed in the Cytoscape display (Figure 9).

The WGCNA R program was used to compute all the genes’ edges and nodes from each of the four modules based on intramodular gene importance. For gene-network visualizations, these data were exported to Cytoscape. The watermelon annotation database was used to extract and annotate genes from all four highly linked modules. Finally, four genes were identified from MEpurple, and among these four genes, two genes, *Cla97C08G155010* and *Cla97C06G128110,* were correlated with IAA (r^2^ = 0.81), one gene, *Cla97C09G169510,* correlated with ZR (r^2^ = 0.85), and one gene, *Cla97C01G002890,* correlated with POD (r^2^ = 0.74). On the other hand, of three genes from the MElightsteelblue1 module, one gene, *Cla97C11G224830,* correlated with CAT (r^2^ = 0.81). Two genes, *Cla97C03G066780* and *Cla97C03G068360,* strongly correlated with SOD (r^2^ = 0.72), while two gene from the MEivory module, *Cla97C07G139710* and *Cla97C04G077300,* strongly correlated with H_2_O_2_ (r^2^ = 0.88). Two genes from the MEdarkolivegreen module, *Cla97C01G023850* and *Cla97C01G006680,* strongly correlated with sugars and starch (r^2^ = 0.87).

### 3.9. RT-qPCR to Verify the Accuracy of Transcriptome Data

**Figure 10:**
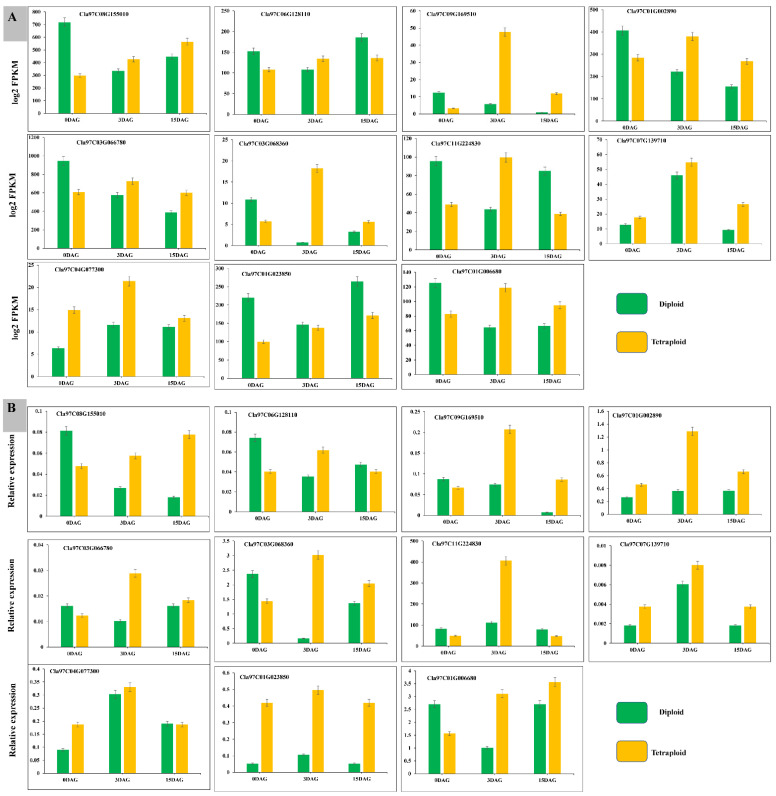
*X*-axis represents the important three grafting phases, 0, 3, and 15 DAG, *Y*-axis represents gene relative expression levels by qRT-PCR and FPKM values by RNA sequencing, green color indicates diploid, and orange represents tetraploid. (**A**) FPKM values by RNA sequencing, (**B**) qRT-PCR relative expressions of chosen genes. Columns indicate mean ± SE.

Precision of transcriptome sequencing data is required for identifying differentially expressed genes and subsequent GO and KEGG function enrichment analyses. To verify the reliability of the transcriptome sequencing data, we selected 11 hub genes from four modules significantly associated with the phenotype to check the relative expressions in diploid and tetraploid plants of watermelon plants at three time points (0, 3, and 15 DAG). At three separate stages, the expressions of genes that regulate CAT, POD, SOD, H_2_O_2_, IAA, ZR, sugars, and starch were examined (0, 3, and 15 DAG). Genes linked to IAA and ZR, *Cla97C08G155010*, *Cla97C06G128110*, and *Cla97C09G169510,* had higher expressions at all three stages in tetraploid watermelons than diploid ones in both seasons. Genes linked to POD, CAT, and SOD, *Cla97C01G002890*, *Cla97C11G224830*, *Cla97C03G066780*, and *Cla97C03G068360,* had a higher expression in tetraploid compared to diploid watermelons at all days after grafting (Figure 10A). Genes regulating H_2_O_2_, *Cla97C07G139710* and *Cla97C04G077300,* had lower expression levels in tetraploid watermelons than diploid watermelons in both seasons. Genes regulating sugars and starch, *Cla97C01G023850* and *Cla97C01G006680,* had higher expressions in tetraploid watermelons than diploid watermelons. Thus, this confirms the active role of these two candidate genes in sugar and starch accumulation at all time points of watermelon sampling. The trends in gene expression evaluated by transcription (FPKM) and relative expression evaluated by the qRT-PCR method were consistent (Figure 10B), indicating that the validation transcriptome data and relative gene expressions confirmed that the key putative genes we selected are true candidates involved in watermelon graft compatibility.

## 4. Discussion

The current study was conducted to compare grafted diploid and tetraploid plants of watermelon plants to study the compatibility mechanisms and genome duplication effects. The obtained data showed that the tetraploid grafted plants have more compatibility than diploid grafted plants with the splice method.

### 4.1. Role of Hormones, AOX and Carbohydrates in the Graft Union during the Grafting Seedling Process in Tetraploid Compared to Diploid Watermelon

Plant hormones play an important central role in callus initiation and vascular bundle formation at grafting unions [40,45,46]. During the grafting process, the maximum IAA contents were observed until 3 DAG in the scion, as reported by Zheng B.S. et al. [77] and Melnyk et al. [78]. Hormonal imbalance and low IAA contents lead to incompatibility [56,79,80]. At various days after grafting, the IAA and ZR contents of diploid and tetraploid watermelon plants in the graft union were compared. With a *p* value of 0.05, the IAA and ZR contents of grafted tetraploid plants were substantially higher than in diploid plants. Our findings revealed that tetraploid plants with high hormone content and increment rates were more compatible than diploid plants, notably at 3 DAG (critical phase) and 15 DAG. These results can explain the higher survival rates in tetraploid than diploid plants; these results were in agreement with Melnyk et al. [78], Cookson et al. [81], and Schaller et al. [82].

According to Gainza et al. [63], incompatibility is caused by stress created during the healing process. POD, SOD, and CAT activities increased much more in tetraploids than in diploids throughout the healing process (Figure 4). Surprisingly, the content of H_2_O_2_ (Figure 4) did not increase during the healing process in tetraploids; these findings emphasized that high antioxidant activities lead to scavenging oxygen radicals and H_2_O_2_. Our results are in accordance with Meng et al. [1] and Fernandez-Garcia et al. [55]. The incompatible grafts showed higher H_2_O_2_ content and lower activity of POD and SOD in the grafting union [54,56,57]. Our findings are in line with those of Jiang et al. [19], Tu et al. [20], Ruiz et al. [21], and Ganie et al. [83], who discovered that genome doubling boosted stress resistance due to increased hormone content and antioxidant activity. Watermelon, rice, citrus, black locust, honeysuckle, kinnow, mandarin, cotton, and rangpur lime have all been shown to be more resistant to abiotic stressors than diploid species [18,19,20,21,22,23,24,25,26]. The grafting process brought more stresses such as injury or wounds, complete dark and high humidity during the healing period [63,84]. The most critical times in the healing process are the 2nd and 3rd days after grafting [78]. The results in both seasons showed significant differences in POD, SOD and CAT activities between tetraploid and diploid watermelons (Figure 4). From our results, we can assume that the increase and decrease in contents of antioxidants played a biovital role during the graft healing process of watermelon.

An increase in starch is said to lead to a boost in survival rates [59,60]. At 0 DAG, tetraploids accumulated considerably more sugars and starch in the graft union than diploids. In comparison to diploids, sugar and starch decrement rates in tetraploids were quite low. These findings might explain why tetraploids outperform diploids in terms of survival rates. Dabirian and Miles [59] and Bartolini et al. [85] both claimed that increased glucose content in grafted tissues might contribute to effective grafting. Hormones and carbohydrates are both important in the process of compatibility and callus formation [59,60,85,86,87].

### 4.2. Transcriptome Sequencing and DEG Screening in Diploid and Tetraploid Plants of Watermelon

RNA-Seq was utilized to investigate transcriptome variations in the grafting union of diploid and tetraploid watermelon plants grafted onto an interspecific squash hybrid, as well as genes involved in the rootstock–scion interaction. At 0, 3, and 15 DAG, DEGs were found in diploid and tetraploid plants. More than 14,586 DEGs were discovered, with over 50 functional categories and metabolic pathways represented. Furthermore, in tetraploids vs. diploids, we observed more DEGs concentrated in biological processes, cellular components, and molecular function, which significantly varied grafting compatibility survival rates. This meant that genes involved in the activities and pathways indicated above were important in the rootstock/scion interaction effect [88]. WGCNA is also a highly useful method, since it allows us to combine phenotypic and transcriptome data to identify critical candidates for transplant compatibility [89].

### 4.3. Role of Genes Related to Hormonal Signaling in Rootstock–Scion Interaction

Auxin may cause the expression of particular genes, known as primary auxin-responsive genes, to be fast and temporary. The Aux/IAA, SAUR, and GH3 gene families are the three primary groups of auxin-responsive genes [90]. In the auxin signal transduction pathways, the Aux/IAA gene family contains important transcriptional repressors. Aux/IAA transcription factors may react to auxin and degrade quickly, triggering the auxin signaling pathway [91,92].

In this work, Aux/IAA gene expression in grafting union was shown to be favorably correlated with tetraploid grafting union survival rates and IAA levels. The foregoing findings suggest that the auxin signaling-mediated grafting compatibility of tetraploid grafted seedlings was aided by the selective expression of genes from the Aux/IAA family.

### 4.4. Expression of Antioxidant Enzymes and Growth-Regulating Factors

Most antioxidant enzyme genes were upregulated in the grafting union of tetraploid plants, whereas practically all antioxidant enzyme genes were repressed in diploid watermelon, according to transcriptome data. Meanwhile, in tetraploid plants, gene expression was shown to be positively correlated with antioxidant enzyme activity. As a result, peroxidases play a role in a variety of physiological processes in plants, including peroxide scavenging, lignification, cell-wall production, and auxin metabolism [93,94]. As a result, considerable antioxidant enzyme gene expression could be produced in grafted plants to boost peroxidase activity. This might speed-up auxin metabolism and the crosslinking of structural proteins, hemicellulose, and pectin in the cell wall, reducing graft compatibility; these findings agree with those of Liu et al. [31].

## 5. Conclusions

In this research, we utilized WGCNA to combine transcriptome profiles and phenotype data to analyze gene networks influencing hormones, AOX, and carbohydrates in diploid and tetraploid watermelon plants (based on co-expression patterns). During the grafting process, four co-expression modules/gene networks were found that were substantially linked with differences in hormone, AOX, and carbohydrate contents. We also found 11 significant candidate genes that were weighted as module hub genes within these networks, and their quantitative expressions were associated with phenotypic data. Multiple stringent screening steps in our study increased the likelihood and confidence that these genes are true candidates in the hormone, AOX, and carbohydrate pathway networks. These genes were discovered recently, and there have been no earlier studies on their roles or functions in watermelon, nor have there been any previous reports on tetraploid grafting.

## Figures and Tables

**Table 1:** RNA sequencing data and corresponding quality control information.

Samples	Di-0DAG	Di-3DAG	Di-15DAG	Tetra-0DAG	Tetra-3DAG	Tetra-15DAG
Clean reads	21,931,767	24,794,003	21,124,267	22,596,008	21,227,314	21,498,313
Clean bases	6,551,749,351	7,401,188,622	6,316,423,223	6,755,177,632	6,352,106,792	6,428,408,569
GC Content	45.54%	44.51%	45.73%	44.85%	44.98%	44.73%
% ≥ Q30	94.15%	93.89%	94.61%	94.93%	94.26%	94.27%
Total Reads	43,863,533	49,588,005	42,248,535	45,192,017	42,454,628	42,996,625
Mapped Reads	18,004,952	23,863,421	12,594,273	34,743,569	22,683,237	22,038,804
Mapped Reads ratio	41.35%	47.58%	29.72%	76.69%	53.17%	52.67%
Uniq Mapped Reads	17,456,213	23,213,051	12,223,916	33,769,354	22,049,895	21,546,888
Uniq Mapped ratio	40.09%	46.28%	28.85%	74.53%	51.69%	51.51%
Multiple Map Reads	548,739	650,370	370,356	974,215	633,342	491,917
Multiple Map ratio	1.26%	1.30%	0.87%	2.16%	1.48%	1.16%
SNP Number	21,441	33,225	27,477	26,737	43,712	45,905

Note: (1) Clean reads: paired-end numbers of clean data; (2) clean bases: total base number of clean data; (3) GC content: GC content percentage of clean data, namely the percentage of clean database G and C; (4) ≥Q30%: the bases whose quality values are greater than or equal to 30% of clean data. (5) Unique Mapped Reads: number of reads mapped uniquely mapped to the reference genome and the percentage in clean reads; (6) Multiple Mapped Reads: number of reads multiply mapped to reference genome and the percentage in clean reads.

**Table 2:** Pairwise comparison of up- and down-regulated DEGs between diploid vs. tetraploid watermelon at 0, 3, and 15 DAG.

DEG Set	DEG Number	Upregulated	Downregulated
Diploid-0_vs_Tetraploid-0	3178	1746	1432
Diploid-3_vs_Tetraploid-3	2002	967	1035
Diploid-15_vs_Tetraploid-15	2457	1232	1225

## Data Availability

Not applicable.

## Supplementary Materials

The following supporting information can be downloaded at: https://www.mdpi.com/article/10.3390/biology11040575/s1, Table S1: Primers used for qRT-PCR in this study.
