# Supplementary material for: Transcriptome Profiling to Dissect the Role of Genome Duplication on Graft Compatibility Mechanisms in Watermelon
Source: Biology (Basel). 2022 Apr 11;11(4):575. doi: 10.3390/biology11040575 (PMC9029962; doi:10.3390/biology11040575)
Supplement: Supplementary file 1 [file biology-11-00575-s001.zip › biology-1659195-supplementary.pdf]

Supplementary data

Original research manuscript submitted to Biology

Title: Transcriptome profiling to dissect the role of genome duplication on graft compatibility mechanisms in watermelon

Authors: Mohamed Omar Kaseb[1,2], Muhammad Jawad Umer[1], Muhammad Anees[1], Hongju Zhu [1], Shengjie Zhao[1], Xuqiang Lu [1], Nan He [1], Eman El-remaly [2] El-Eslamboly A.A.S.A[2], Ahmed F. Youesf[3], Ehab A.A. Salama[4], Abdulwahed Fahad Alrefaei[5], Hazem M. Kalaji[6,7] and Wenge Liu[1,*]

1 Zhengzhou Fruit Research Institute, Chinese Academy of Agricultural Sciences, Henan Joint International Research Laboratory of Fruits and Cucurbits Biological Science in South Asia Zhengzhou 450009, China; umermjawad@yahoo.com (M. J. U.), aneesgscaas@outlook.com (M. A.), zhuhongju@caas.cn (H. Z.), zhaoshengjie@caas.cn (S.Z.), luxuqiang123@163.com (X. L.), henan@caas.cn (N. H.)
2 Horticulture Research Institute, Agriculture Research Center, Giza, 12119, Egypt; mohamedkaseb@yahoo.com (M. O. K,); emanelrmaly@yahoo.com (E. E); azaz2005asd@yahoo.com (E. A.A. S.)
3 Department of Horticulture, College of Agriculture, Al-Azhar University (branch Assiut), Assiut 71524, Egypt; ahmed.yousuf@azhar.edu.eg (A.F.Y.)
4 Agricultural Botany Department, Faculty of Agriculture (Saba Basha), Alexandria University, 21531 Alexandria, Egypt; ehabsalama89@alexu.edu.eg (E. A. A. S.)
5 Department of Zoology, College of Science, King Saud University, P.O. Box 2455, Riyadh 11451, Saudi Arabia; afrefaei@ksu.edu.sa (A. F. A.)
6 Department of Plant Physiology, Institute of Biology, Warsaw University of Life Sciences SGGW, Warsaw, Poland; hazem@kalaji.pl (H. M. K.)
7 Institute of Technology and Life Sciences, National Research Institute, Falenty, Al. Hrabska 3, 05-090 Raszyn, Poland; hazem@kalaji.pl (H. M. K.)
* Correspondence: author: e-mail: liuwenge@caas.cn (W. L.)

**Primers used for qRT-PCR in this study.**	
ID	primer
Actin-F	GTACGACAACGGGCCTTAAA
Actin-R	ATGGGCTTGACAGGTTGTTC
WMCAT-1-F	CTAATGTGTTGAGCGGCAAA
WMCAT-1-R	GTCTTACGAACCGCTCTTGC
WMCAT-2-F	CGGCATTACTAAAGCGGAAG
WMCAT-2-R	TAGTCCAGAAGGGGGTGTTG
WMPOD-1-F	TTGGATGCCTACACGATCAA
WMPOD-1-R	GTATTGGCCAGCTTCACCAT
WMPOD-2-F	CATTGGGAAGGAAGGACTCA
WMPOD-2-R	CAATGTCAAGCCCTTGGTTT
WMPOD-3-F	GCTCTTTCAGGAAGCCACAC

WMPOD-3-R	GGTAGGGCTCACAAAGTCCA
WMPOD-4-F	TGGCTGTGATGCTTCTATGC
WMPOD-4-R	CTGCACAAGAGACGATTCCA
WMSOD-1-F	TTTCTAAGGTCCACCGCAAC
WMSOD-1-R	GGGAATTGGGTTTGAAGGAT
WMSOD-2-F	CAAGAAGACGATGGAGCACA
WMSOD-2-R	TCCATCAGCATTGGCAATTA
WMSOD-3-F	CCATCCAGTTCGTCCAAGAT
WMSOD-3-R	CCTCCATGGTCCTTCTTCAA
WMH2O2-1-F	GGGTTACTTCGGACCAGACA
WMH2O2-1-R	GAAACCCGTCAGCCTATCAA
WMH2O2-2-F	ATGACCAAAAACGTCGGAAG
WMH2O2-2-R	ACTCGCTCGACTCGTTCATT
WMH2O2-3-F	AACTTCTGCTTCGACCTCCA
WMH2O2-3-R	AACTCGTGCGAGAAATGCTT
WMH2O2-4-F	AGCCAACAAACGAAACCAAC
WMH2O2-4-R	TCTGTGGCGATATTGGATGA
WMH2O2-5-F	CAGTCCAGGTTCCATTCGTT
WMH2O2-5-R	TTCTCAACGCGAACTCCTTT
WMIAA-1-F	AATGTCGAGTGGCCGATAAC
WMIAA-1-R	GCACCGTGGTGCCTTATACT
WMIAA-2-F	TTCCCACGCTGGAATTTTAC
WMIAA-2-R	GGGAGGAATTCGAAGTAGCC
WMIAA-3-F	ACCTCTATGTGCCTGGGTTG
WMIAA-3-R	GGCAAAGAATGGCCTCATTA
WMIAA-4-F	GGTGAATTTGAAGCCGATGT
WMIAA-4-R	CAAGCTCCTTTTGCAACTCC
WMIAA-5-F	AACAATTCCAGGCCACTACG
WMIAA-5-R	CCCTGTCGGTATACGCTGTT
WMZR-1-F	GACCGCCTATTGCCTCATTA
WMZR-1-R	TTCCCACACACATTGCTGTT
WMZR-2-F	CGCATTATCCCAGCTCTCAT
WMZR-2-R	TAGCGAGACTGAGCGAGACA
WMZR-3-F	GTTAGCGAGGGGAGCTTTCT
WMZR-3-R	AGCACTCTTTTTGGCGTTGT
WMZR-4-F	GTGTCGTGGACATGTTGGAG
WMZR-4-R	GCTAGAGCCAAATGCTGTCC

WMZR-5-F	GTTACGCGGGGTATATGGTG
WMZR-5-R	GCTGCTTTCAAACTCGGTTC
WMLignin-1-F	CCTTTATGGGGGAATCCTGT
WMLignin-1-R	GTGAACAAAAACGGGTTGCT
WMLignin-2-F	ACTCGTTGCCTTTGTCTCGT
WMLignin-2-R	GAAACTGCAACTCGTGCAAA
WMLignin-3-F	TATGGAAAAGGATGGGTGGA
WMLignin-3-R	CTTTAACGTGGCTCGTCCTC
WMLignin-4-F	GAGTCGGAGGTTTGGTGTGT
WMLignin-4-R	CCGACCAAGAGATGAGAAGG
WMPhenols-1-F	GGCTCATAAATCCCCCATTT
WMPhenols-1-R	TGCAGGTTGTTGTCCAGTGT
WMPhenols-2-F	CTCACTATGTCGCCGGATTT
WMPhenols-2-R	ACCGTGAAGGTGGAGAGATG
WMPhenols-3-F	AAGGTGCCTGCTTCACCTAA
WMPhenols-3-R	TATTTCCCCAACCAAACCAA
WMPhenols-4-F	CGATTGAGCAACAAAAAGCA
WMPhenols-4-R	CTGTAGGAGGGCTCACGAAC
WMPhenols-5-F	CCCGATACTCATCGTTCGTT
WMPhenols-5-R	GGCGTAGAAACCCAGGTACA
WMStarch-1-F	TGATTGGCACACAGCTCTTC
WMStarch-1-R	AATTGATCGGGCAGATTCAG
WMStarch-2-F	GCAAAATTTTCCCGAGATGA
WMStarch-2-R	TTTTATTCTCGCACGCCTCT
WMStarch-3-F	GCTCTGGCTTTGGTTCTTTG
WMStarch-3-R	GTCGCATTTTACACGAAGCA
WMStarch-4-F	TTTGATCATGGGAGCATTCA
WMStarch-4-R	GCCTCTTTGGGAACATGAAA
WMStarch-5-F	AAAGGACGGACAACCAAGTG
WMStarch-5-R	GCCAGCTACGTCTCCAAGTC
